# 433. COVID-19 Vaccination Uptake Among Health Care Workers (HCW) Requesting Medical Exemptions to Vaccination.

**DOI:** 10.1093/ofid/ofac492.508

**Published:** 2022-12-15

**Authors:** Jana Shaw, Loren Brown, Stephen J Thomas, Lynn M Cleary, Jarrod Bagatell

**Affiliations:** Upstate Medical University, Syracuse, New York; Cornell, Ithaca, New York; Upstate Medical University, Syracuse, New York; Upstate Medical University, Syracuse, New York; Upstate Medical University, Syracuse, New York

## Abstract

**Background:**

New York State adopted a COVID-19 vaccination requirement for all healthcare workers in September 2021, but they allowed medical exemptions. We examined reasons and frequency of medical exemption requests in an academic medical center.

**Methods:**

We conducted active surveillance of all medical exemption requests in a tertiary care academic center in Central NY. Age, gender, reason for request, prior acceptance of other required vaccines, letter of support from employee provider, adjudication of the request, and impact of the decision on COVID-19 vaccine acceptance were collected prospectively since the mandate became effective.

**Results:**

Among 8,776 HCWs, 108 requested medical exemptions, among those 57 (53%) were denied, 39 (36%) were granted temporary exemption, and 12 (11%) were permanent (Table). Females were more likely to request medical exemptions compared to males, 92 (85%) versus 16 (15%), respectively. Overall, 94 (87%) of the HCWs had a letter from their provider in support of their exemption. Nevertheless, only 47% of those qualified for permanent or temporary exemption using CDC guidelines. The most common reasons for requesting exemption included: having natural immunity, receiving monoclonal antibodies, experiencing a common reaction to previous COVID-19 vaccination, and having an underlying medical condition (Figure 1). The majority of individuals who had a request denied or who received a temporary medical exemption were subsequently vaccinated, 63% and 79%, respectively (Figure 2).

Demographic and other characteristics among health care workers (HCW) requesting medical exemptions.

**Table:**
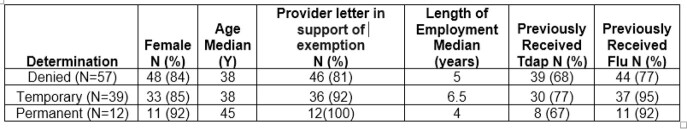

Reasons for requesting medical exemption from COVID-19 vaccination requirement by request determination.

**Figure 1:**
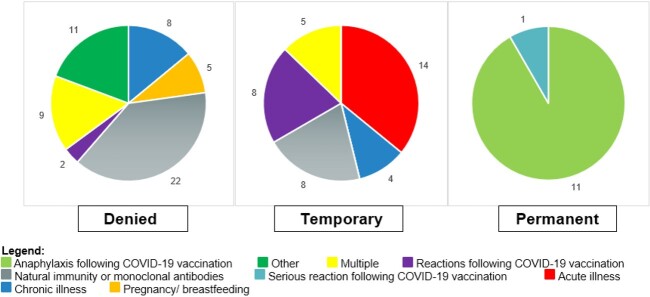

COVID-19 vaccine uptake among health care workers (HCW) requesting medical exemptions to COVID-19 vaccination. *One HCW received alternative COVID-19 vaccine.

**Figure 2:**
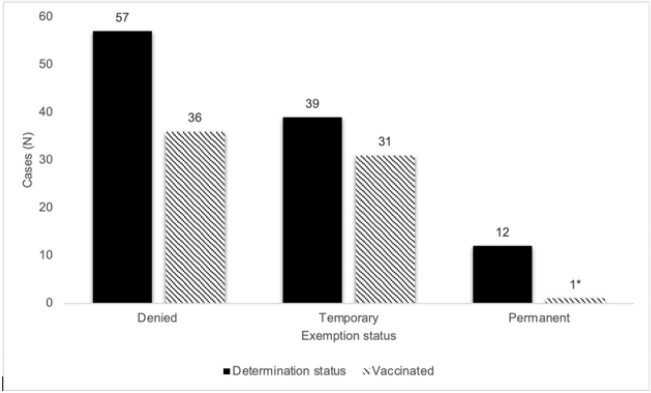

**Conclusion:**

Females were more likely to request a medical exemption to vaccination and their requests were often supported by their providers. Future efforts should focus on educating both health care providers and the public about actual medical contraindications or precautions to vaccination to improve overall vaccination rates.

**Disclosures:**

**Jana Shaw, MD,MS,MPH**, Pfizer: Advisor/Consultant **Stephen J. Thomas, MD**, Clover: case adjudication committee (compensated for time)|EdJen: Advisor/Consultant|Icosavax: data monitoring (compensated for time)|Island Pharma: Ownership Interest|Merck: Advisor/Consultant|Moderna: chair, safety monitoring committee (compensated for time)|New Day Diagnostics: Honoraria|Pfizer: Advisor/Consultant|PrimeVax: Ownership Interest|Sanofi Pasteur: Advisor/Consultant|Takeda: Advisor/Consultant|Takeda: case adjudication committee (compensated for time)|Vaxxinity: data monitoring committee (compensated for time).

